# An empirical analysis of the supply chain flexibility using blockchain technology

**DOI:** 10.3389/fpsyg.2022.1004007

**Published:** 2022-09-26

**Authors:** Mengmeng Wang, Yang Yang

**Affiliations:** College of Business, Gachon University, Seongnam, South Korea

**Keywords:** blockchain technology, digital leadership, supply chain flexibility, supply chain trust, supply chain management

## Abstract

Building a flexible supply chain can enable the firms to manage their supply chains to adapt effectively to dynamic market demand changes and thus guarantee their accelerated growth in the future. In this vein, this study aims to address several important issues in supply chain management by considering two characteristics of blockchain technology (i.e., information transparency and security of blockchain technology) and exploring the specific conditions under which firms are likely to develop trust in supply chain management. Furthermore, we argue that such supply chain trust is vital to the success of achieving and increasing supply chain flexibility. In addition, we propose that top management teams’ digital leadership within the firms plays a vital role in moderating the contribution of each dimension of blockchain technology to supply chain trust. Using data from a large sample of 338 firms in China, we perform structural equation modeling to examine our conceptual framework empirically. Our results highlight and support the idea that blockchain technology’s information transparency and security influence the trust-building in a supply chain and supply chain flexibility and articulate the particular importance of digital leadership in explaining the contribution of different blockchain technology characteristics to trust-building. Our study advances the theoretical, empirical, and managerial analysis of critical factors to build trust and achieve flexibility in supply chains.

## Introduction

The global supply chain relationship has been affected by economic globalization and the epidemic and is thus facing many challenges. The internal and external environments firms face constantly change, and the traditional supply chain model has gradually become fragile and unable to meet the growing needs of firms (Sarkis et al., 2020). To cope with the ever-changing needs of consumers and environmental uncertainty and survive in the fierce market competition environment, firms need to work together to solve the diverse needs of customers. How to build supply chain trust has become an urgent problem to be solved in academia. Supply chain trust is critical (Kshetri and Voas, 2019). Once partners in the supply chain face a crisis of trust, sensitive data and information required for cooperation cannot be shared in time, which can easily lead to a bullwhip effect (Mejia et al., 2019). Supply chain trust can reduce search transaction costs and opportunism (Bierly and Gallagher, 2007). High trust partnerships can reduce coordination costs and enhance knowledge sharing and information flow between cooperative firms (Neeley and Leonardi, 2018). Supply chain trust helps firms build a flexible supply chain that can improve the ability to deal with uncertainty and allow firms to respond quickly to form a core competitive advantage and meet market needs more flexibly and in a targeted manner (Baah et al., 2022). A flexible supply chain means that the supply chain built by a firm can immediately respond to customer needs (Slack, 2005; Roberta et al., 2014). It can deal with conflicts quickly during strategic decision-making and improve the responsiveness and agility to environmental changes to cope better with unforeseen situations, avoid delivery delays and customer dissatisfaction, respond to consumer demand with other firms quickly in the supply chain, and maintain inventory at a controllable level (Swafford et al., 2008). In other words, to respond to environmental changes and provide customer-oriented products and services, firms need to build flexible supply chains to seize opportunities and reduce risks (Tallon and Pinsonneault, 2011; Gligor, 2013). Building a flexible supply chain can also help firms improve their efficiency and competitiveness; in a changing technological environment, flexible supply chains can provide customers with customized products and services (Delic and Eyers, 2020). Although the existing literature has emphasized the benefits of building a flexible supply chain, empirical analysis on how to build a flexible supply chain is lacking. Thus, the present study’s first major aim is to understand the key driving forces and influencing factors of building a flexible supply chain through empirical analysis.

Supply chain trust is necessary for building an effective supply chain relationship. The crisis of trust seriously affects the relationship between firms in the supply chain. Supply chain trust helps firms to share information, benefits, and risks. Establishing supply chain trust between firms is always challenging (Stonkutė and Vveinhardt, 2016; Song et al., 2019). In particular, whether the information and communication technology represented by blockchain can provide support for supply chain trust has attracted much attention. Some scholars have advocated that blockchain technology is one of the disruptive frontier technologies in the fourth industrial revolution and plays a vital role in the realization of information resource sharing and trust for firms (Queiroz et al., 2019; Saberi et al., 2019; Wamba et al., 2020). Blockchain technology can promote supply chain trust through digital signatures, smart contracts, and the immutability of transaction records (Dubey et al., 2020; Ji et al., 2022; Wan et al., 2022). However, some scholars have advocated that the use of blockchain technology in the supply chain needs to consider the traceability awareness of consumers and the cost of adopting blockchain technology (Fan et al., 2022). Given the shortcomings of blockchain technology, such as limited throughput, time consumption, low security, and limited storage space, t whether it can bring value to business processes remains uncertain. Therefore, some scholars are on the sidelines of adopting blockchain technology and even refuse to adopt blockchain technology (Swan, 2015; Liang et al., 2021). Furthermore, blockchain technology is not yet mature and has many scalabilities, performance, and compatibility problems with other systems; thus, firms are facing huge management challenges (Lacity, 2018). In summary, different scholars have varying opinions on whether blockchain technology is suitable for the supply chain environment. The characteristics of blockchain technology need to be subdivided for profound research. In this study, blockchain technology is divided into two core characteristics: information transparency and security. Through empirical analysis, the relationship between the two characteristics of blockchain technology, supply chain trust, and supply chain flexibility, is clarified to fill the gap in relevant research.

Furthermore, according to previous literature, the disruption brought about by digital technology requires companies to play the role of digital leadership (Westerman et al., 2014). Businesses with digital leadership can develop a clear digital strategy (Zeike et al., 2019). Only digital leadership can change the behavior of leaders and the organizational structure of companies to support digitally enabled business models (Oberer and Erkollar, 2018). Thus, this study uses digital leadership as a moderating variable to explore the moderating role of digital leadership in the relationship between information transparency and security of blockchain technology and supply chain trust. Valuable suggestions are suggested for firms to improve the supply chain and help them win first place in future supply chain competition.

This study provides three key contributions to the blockchain technology and supply chain management research amidst the backdrop of the COVID-19 pandemic situation. As one of the most potent disruptive information technologies and innovations sweeping the world today, blockchain technology has great potential to deal with a recent special issue on supply chain trust between firms. This study makes an important theoretical contribution by addressing a relatively neglected issue: the impact of specific blockchain technology characteristics (i.e., information transparency and security) on supply chain trust and flexibility. In addition, we also contribute to the literature by enabling an evaluation of the moderating role of digital leadership in the relationships between information transparency, security, and supply chain trust. Finally, our study makes an empirical contribution by testing these relationships. Our empirical results offer novel insights into the strategic management of blockchain technology, and trust and flexibility in the supply chain.

The remainder of the paper is organized as follows. In the next section, we provide the theoretical background and develop a series of research hypotheses. We then describe the research method employed to test the proposed hypotheses. In the following section, we present the results of the empirical analysis. Finally, we discuss the findings and their implications.

## Theoretical background and hypotheses development

The main emphasis of the network theory is to understand and explain the nature and interactions of a firm’s external networks which involve relationships with various parties, such as customers, suppliers, competitors, and other actors with which the firm is connected (Gulati et al., 2000). The network theory has become increasingly a useful tool for diagnosing network relationships in management consulting (Borgatti and Halgin, 2011) and explaining why firms should learn to work effectively with their network partners by building high-quality trust-based relationships (Afuah, 2013). To obtain external resources, firms need to establish a stable cooperative relationship, making the network between firms more stable (Treiblmaier, 2018). Network relationships are expected to facilitate the rapid internationalization of blockchain startups (Zalan, 2018). One valuable outcome for the firms from the building of trusting relationships with their partners is that such trust-based relationships may represent an opportunity for the firms to access needed strategic assets, such as information, knowledge, and other important resources (Davis et al., 2000; Hitt et al., 2001). Blockchain technology can help firms and their partners build trusting relationships and thus promote mutual trust in the changing process of business relationships (Tian, 2016). Blockchain technology mainly focuses on generating trust between firms through cooperative relations and interactive processes. From a blockchain perspective, network theory can be employed to help assess how networks among businesses interact. Partnerships and information transparency between businesses can help managers understand whether personal relationships can be replaced by the exchange of information provided by blockchain technology (Kummer et al., 2020).

**FIGURE 1:**
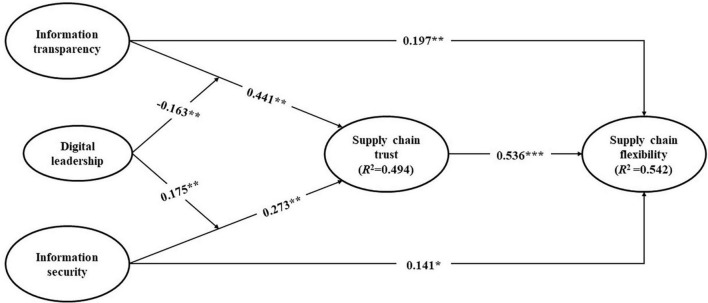
Conceptual framework.

To gain a competitive advantage, the resource-based view (RBV) emphasizes that firms need to have rare, valuable, and irreplaceable resources, which are difficult to imitate (Barney, 1991, 2001; Singh and Samuel, 2020). The resource coordination ability to use internal and external key resources to acquire information technology plays an important role in the acquisition of resources and the improvement of corporate performance (William et al., 2013). RBV is widely used in companies’ digital transformation using the fourth industrial revolution digital technology (Bordeleau et al., 2020). Blockchain technology is one of the digital technologies at the forefront of Industry 4.0. Building on the network theory and RBV, the present study proposes a conceptual framework which is depicted in Figure 1. More specifically, we examine a central research question: how may blockchain technology contribute to building supply chain trust? We theorize and examine this central question by dividing blockchain technology into two dimensions: information transparency and security of blockchain technology. As a foundational issue, we first develop and test hypotheses to suggest that the information transparency and security of the blockchain may have a positive impact on supply chain trust. Second, we consider the respective influence of the blockchain’s information transparency and security on the flexible supply chain. Third, we theorize and examine whether digital leadership plays a vital role in moderating the effect of blockchain information transparency and information security on supply chain trust. Finally, we theorize and examine how supply chain trust may contribute to supply chain flexibility.

### Blockchain technology information transparency and supply chain trust

Supply chain trust is the opposite of opportunism and reflects the degree of interdependence among supply chain partners (Capaldo and Giannoccaro, 2015). Partners with supply chain trust will abide by the commitments of both parties and will not increase their interests at the expense of the interests of both parties (Cao and Zhang, 2010). Given the deepening of economic globalization, the business network of firms has become increasingly complex, and supply chain trust is crucial in the interaction process of completely unfamiliar firms (Xu et al., 2021). Information transparency refers to the willingness and behavior of companies to disclose information (Turilli and Floridi, 2009). Firms can easily confirm historical transaction records and ensure that members in the supply chain need to notify other members for approval when changing and deleting information (Kim and Shin, 2019). Problems such as poor information flow, difficult logistics tracing, and untrue capital flow often occur during supply chain operations. Blockchain technology is expected to bring opportunities to improve these problems (Hu et al., 2021; Wu and Zhang, 2022). The increased utilization of blockchain technology can increase trust among suppliers (Meidute-Kavaliauskiene et al., 2021). Blockchain technology can help firms in the supply chain to be more transparent in information flow, logistics, and capital flow to strengthen the trust between supply chain partners (Wu and Zhang, 2022). Traditional supply chain firms heavily rely on central institutions or third parties to promote the trust between participants in the cooperation process. However, relying on a third party can easily cause malicious attacks and tampering. Blockchain technology facilitates the maintenance of key information through its distributed and decentralized characteristics, and each node has a copy of transaction data, thereby improving the transparency of business activities and establishing a foundation for supply chain trust (Chang and Chen, 2020). After using blockchain technology, the information can be traced, and the transaction information is not easily tampered with. A problem can be investigated for responsibility through information tracking, which effectively alleviates the goal conflict between suppliers, thereby increasing the trust of the supply chain (Pournader et al., 2020). In summary, the transparency of blockchain technology can enhance supply chain trust. Thus, this study proposes the following hypothesis:

*Hypothesis 1(H1)*: The information transparency of blockchain technology has a positive effect on supply chain trust.

### Blockchain technology information security and supply chain trust

Blockchain technology enables secure data exchange in a distributed manner, improving firm supply chain operations (Ghode et al., 2020). Calibration of end-to-end data across various supply chains cannot be achieved without blockchain, which enhances the security of stored data and ensures real-time access to all information (Dutta et al., 2020). Blockchain technology enables monitoring of the compliance status of individual suppliers while keeping sensitive documents safe (Venkatesh et al., 2020). Decentralization ensures that data cannot be changed in any way. Encryption technology ensures data security while safeguarding that data cannot be changed without knowing the correct key. Consensus protocols secure the entire network by requiring all nodes to have a unified protocol (Lim et al., 2021), and the adoption of blockchain technology by firms can increase trust in customers (Dutta et al., 2020). Furthermore, blockchain technology has the immutability of data, which ensures information security and helps increase users’ trust in the entire supply chain transaction (Rejeb et al., 2021). Smart contracts of blockchain technology have an encryption system to ensure a safe user experience, and smart contracts with pre-determined terms remove human judgment from transactions and reduce human risk. After adopting blockchain technology, transaction records are encrypted, considerably reducing the possibility of network attacks and improving the payment security between upstream and downstream customers. Blockchain technology can help consolidate supply chain partnerships (Kim and Shin, 2019). When the supply chain partnership becomes more consolidated, supply chain trust will also greatly improve. Based on this, this study proposes the following hypothesis:

*Hypothesis 2(H2)*: The information security of blockchain technology has a positive effect on supply chain trust.

### Blockchain technology information transparency, security, and flexible supply chain

Building a flexible supply chain can enable firms to deal with supply chain disruptions and demand changes effectively (Fischer et al., 2016). Supply chain members need to enhance their flexibility to improve their ability to respond to market changes (Moon et al., 2012). In the current market competition, firms often outsource some business processes to improve the flexibility of the supply chain (Hald and Kinra, 2019). When outsourcing part of the business, only by allowing information to be effectively shared among firms can the supply chain be flexible (Manders et al., 2017; Meidute-Kavaliauskiene et al., 2021). Given that blockchain technology is based on a database, all stakeholders involved in the network use blockchain technology to store and distribute data, which can ensure the security and transparency of information and make the sharing of information resources between firms in the supply chain more secure and reliable (Queiroz et al., 2019; Saberi et al., 2019; Lohmer et al., 2020). At the same time, using blockchain technology can also track product trends and help firms make adjustments quickly. The applications used and introduced can only be implemented at relevant network points, thus ensuring data security and transparency during supply chain transactions (Wang et al., 2020). Furthermore, blockchain technology can also help firms realize real-time data sharing on the network, which helps firms to respond flexibly and resiliently when uncertainty occurs (Lohmer et al., 2020). Given that flexible response and elasticity are important factors for firms to build a flexible supply chain, the information transparency and security of the blockchain are expected to play a positive role in promoting firms to build a flexible supply chain. On this basis, the following hypotheses are proposed:

*Hypothesis 3(H3)*: The information transparency of blockchain technology has a positive effect on supply chain flexibility.

*Hypothesis 4(H4):* The information security of blockchain technology has a positive effect on supply chain flexibility.

### The moderating role of digital leadership

Given the volatility, uncertainty, and complexity of the digital economy, managers must have digital leadership (Petry, 2018). Digital leadership is a leadership approach designed to support firms in implementing digital business models by changing leaders’ behaviors, organizational structures, and workforce management. Firms with digital leadership are more likely to stick out in the fourth industrial revolution (Oberer and Erkollar, 2018). Digital leadership is a complex concept. First, managers must change their roles, skills, and leadership styles. Second, digital organizations should be built at the levels of governance, vision, values, culture, and decision-making process. Finally, staff management, knowledge, communication, and collaboration should be adjusted at the personal level. Digital leadership aims to build a customer-centric digital business model (Eberl and Drews, 2021). Managers with digital leadership are visionaries; they are proficient in digital technology and like to motivate employees to innovate and adopt new technologies to become digital experts (Promsri, 2019). Managers with digital leadership understand digital technology skills themselves, use digital thinking to make decisions, and can adequately assess digital-related opportunities and challenges (Hensellek, 2020). At the same time, managers with digital leadership like to use platforms such as social media to engage with customers, partners, employees, and other stakeholders across the company, thereby increasing interaction and trust (Bennis, 2013; Yücebalkan, 2018). As one of the cutting-edge digital technologies in the fourth industrial revolution, blockchain technology can predict that managers with stronger digital leadership are more willing to use the transparency of blockchain technology to promote supply chain trust. Digital leadership emphasizes the need for secure governance of data privacy and information quality (Outvorst et al., 2018). Managers with strong digital leadership have strong digital skills to identify the opportunities and risks associated with digitalization, especially the security of digital devices, software, data, and digital behaviors (Crummenerl and Kemmer, 2015). In other words, firms with strong digital leadership pay more attention to information security. Firms with digital leadership are likely to use the security of blockchain technology to promote supply chain trust. Thus, the following hypotheses are proposed:

*Hypothesis 5(H5)*: Digital leadership positively moderates the effect of information transparency of blockchain technology on supply chain trust.

*Hypothesis 6(H6)*: Digital leadership positively moderates the effect of information security of blockchain technology on supply chain trust.

### Supply chain trust and flexible supply chain

Firms can handle different non-standardized orders by establishing flexible supply chains, providing customized products for customers, producing products of different sizes and colors, and adjusting production in time according to the needs of customers and target markets (Al-Shboul et al., 2017). A flexible supply chain can improve the responsiveness and agility to environmental changes, respond to consumer needs with other firms in the supply chain quickly, avoid delivery delays and customer dissatisfaction effectively, and maintain inventory at a controllable level (Swafford et al., 2008), reducing uncertainty and risks in the supply chain. The firms can quickly respond to sudden disruptions and changes in the supply chain (Liao, 2020; Katsaliaki et al., 2021; Sundgren, 2022). Building a flexible supply chain cannot be completed by one firm alone. It needs the participation of all firms in the supply chain. Firms must reach the supply chain trust before they become willing to share information actively to avoid the risk of uncertainty (Ghode et al., 2020). The trust relationship between partners can make the firm supply chain management achieve better performance. The mutual trust cooperation relationship can ensure that all parties in the supply chain understand one another’s business and dynamics and are willing to assist in developing innovative solutions. Without trust as a foundation, cooperative alliances can neither be established nor sustained (Batwa and Norrman, 2021). Supply chain trust can also affect the resilience of firms in the supply chain network (Hou et al., 2018; Delic and Eyers, 2020). Given that elasticity is important in building a flexible supply chain, supply chain trust is expected to promote firms to build a flexible supply chain. Thus, this study proposes the following hypothesis:

*Hypothesis 7(H7)*: Supply chain trust has a positive effect on supply chain flexibility.

## Methodology

### Sampling and data collection

We empirically examine our research hypotheses by collecting data from a large sample of firms in China. We believe China offers an ideal setting owing to the following three reasons. First, China has become one of the world’s most important economies in driving the development of blockchain technology and applications across different sectors. Blockchain technology has been quickly and widely applied in many areas in China, such as finance, medicine, government data sharing, and supply chain management. With its large-scale market and significant efforts to accelerate expansion in the blockchain industry, China is expected to occupy a more advantageous position relative to other developed or emerging economies in testing blockchain technology, thus leading blockchain technological innovation and application globally. Second, the Chinese government has played an important role in China’s blockchain technological development and adoption. According to a guideline jointly released in 2021 by the Ministry of Industry and Information Technology and the Office of the Central Cyberspace Affairs Commission, China aims to enhance its efforts to boost further the technological application and industrial development of blockchain in the next decade and become a globally competitive player in the blockchain industry by 2025. In particular, China also aims to integrate blockchain deeply with other next-generation information technologies, such as big data and artificial intelligence in China, by nurturing several globally competitive firms, several innovative firms related to blockchain, and several blockchain industrial parks by 2030. Third, Chinese firms are currently leading in developing and adopting blockchain technology in terms of global blockchain patent filings and developing blockchain projects. According to the data reported by Tianyancha, a corporate information provider, approximately 121,000 firms are operating in blockchain-related industries. Chinese firms have filed the most patents related to blockchain in the world, including some of the biggest names in blockchain, accounting for more than two-thirds of the world’s blockchain-related patents. As reported by Blockdata, China also led the world in blockchain projects initiated, with 786 blockchain projects in progress, which accounted for more than 48.5% of the global total blockchain projects in 2018. Finally, China is emerging as one of the most significant economies in adopting blockchain technology to boost and stabilize its global trade amid the COVID-19 pandemic. For example, China has successfully adopted blockchain technology to lower logistics costs through the China-Europe train routes in southwest China’s Sichuan Province. Using the blockchain-powered platform Sino-Europe Trade Link 2.0 by the Industrial and Commercial Bank of China, international trade firms can raise funds directly from the bank and thus achieve efficiency and effectiveness by lowering costs and speeding up the cash flows.

We collected the primary data through a survey approach in China. We carefully developed a well-structured survey questionnaire. We first developed an English-language questionnaire and then translated it into Chinese with the assistance of two independent bilingual translators. Finally, the Chinese survey questionnaire was back-translated into English by two other independent bilingual translators to ensure conceptual equivalence and accuracy. Prior research has argued about the potential difficulties in collecting sufficient and reliable primary data from firms in China (Hoskisson et al., 2000) and has pointed out the particular importance of utilizing trust-based relationships to obtain effective, valid, and reliable data in the Chinese market. Thus, we hired a national research company in the local Chinese market to help us administer and process the survey. Through such well-structured survey procedures, we received 351 questionnaires. After omitting 13 incomplete responses, we received 338 completed and usable effective responses, which were used in our final data analysis.

We checked for the potential concerns about non-response bias that will likely arise in survey-based data. We compared the differences between the responding and non-responding firms and the early- and late-responding firms in terms of key firm characteristic variables (e.g., firm size) to assess the possibility of non-response bias in our data. We did not find any statistically significant differences between these groups, suggesting that non-response bias is less likely to occur in the study. We also assessed the possible presence of common method variance (CMV). Owing to the efforts made to develop our well-structured questionnaire and administer the survey, we were quite confident that CMV was less likely to occur in our data. Nevertheless, we assessed the possible occurrence of CMV in our data by following the procedures recommended by Podsakoff et al. (2003). More specifically, we performed Harman’s one-factor analysis using exploratory factor analysis with all multiple-item scales being entered into a non-rated factor analysis. The results of the exploratory factor analysis suggested that no general factor is apparent in the unrotated factor structure and accounts for a majority of the variance, providing no evidence of serious CMV problems in the data.

### Variables and measurement

Unless indicated otherwise, all the dependent and independent variables were measured with multiple-item, seven-point Likert scales ranging from “strongly disagree” (1) to “strongly agree” (7).

In this study, we asked the firm to assess its overall capability to respond to unexpected changes in the dynamic business environment and evaluate the degree of a firm’s supply chain flexibility (Srinivasan and Swink, 2017; Gupta et al., 2019). Following prior research (Cao and Zhang, 2010; Jin et al., 2014; Sreedevi and Saranga, 2017; Gupta et al., 2019), we measured the firm’s supply chain flexibility using seven items. We used nine items adopted from prior studies (e.g., Cao and Zhang, 2011; Capaldo and Giannoccaro, 2015) to capture the degree of a firm’s supply chain trust. We adopted four items derived from prior studies (e.g., Kim and Shin, 2019; Queiroz and Wamba, 2019; Malik et al., 2021; Xu et al., 2021) to measure the information transparency of blockchain technology. Similarly, following prior research (Moody et al., 2018; Kim and Shin, 2019; Xu et al., 2021), we used four items to measure the degree of information security of blockchain technology. We adopted five items derived from previous research (e.g., Chen and Chang, 2013; AlNuaimi et al., 2022; Erhan et al., 2022) and modified them to fit the context of blockchain technology transformation.

Furthermore, we also included several control variables in the estimation to control for alternative explanations for the results: firm size, firm age, and experience in blockchain technology adoption. We included firm size in the analysis, measured as the number of total employees of a firm, to control for the confounding effect of firm size (Dehghani et al., 2022). We included firm age, measured as the number of years since the firm’s founding, to control for the effect of firm age on new technology adoption (e.g., Verhoef et al., 2021). Finally, we included a firm’s experience using blockchain technology by creating a dummy variable equal to 1 if the firm had any experience in using blockchain technology.

## Analyses and results

### Measure reliability and validity assessment

We used the partial least squares (PLS) structural equation modeling (SEM) approach to test our proposed model empirically (Sinkovics et al., 2015). Before testing the proposed hypotheses, we first assessed the reliability and validity of the constructs used in the study. Table 1 presents the results of the reliability and validity assessment. We measured reliability by calculating Cronbach’s alpha and composite reliability for the construct measures, which are wide reliability measures (Nunnally, 1978; Fornell and Larcker, 1981). Table 1 shows that all the Cronbach’s alpha and composite reliability values are greater than 0.80, suggesting an adequate level of reliability and validity for all the construct measures adopted in the study. Furthermore, the factor loading of all constructs is highly significant and greater than 0.70, demonstrating the strong reliability of our measurement model (Chin, 1998). We calculated each construct’s average variance extracted (AVE) values to assess convergent validity. The results presented in Table 1 documented that all AVE values are greater than 0.50, suggesting adequate convergent validity and reliability of the measures (Fornell and Larcker, 1981). Moreover, following the procedures recommended by Fornell and Larcker (1981), we evaluated discriminant validity by comparing each construct’s square root of AVE with the correlation between the construct and other constructs in the model. As reported in Table 2, the results revealed that each construct’s square root of AVE is greater than the correlation between the construct and others, exhibiting a strong discriminant validity of the measures for all constructs. We also checked for the loading values of every single indicator with the cross-loadings with other ones to assess the discriminant validity of the measures. The results showed that each indicator loading is higher than the respective cross loading, providing additional evidence of adequate discriminant validity of the measures employed in the study. Furthermore, we checked for the heterotrait-monotrait ratio (HTMT) of the correlations. We found that all HTMT correlations values are not higher than 0.85, again offering evidence of satisfactory discriminant validity for all measures included in the model (Henseler et al., 2015). Finally, we assessed the predictive validity of the latent constructs in the model using Stone–Geisser’s Q2 recommended by prior research (e.g., Stone, 1974; Geisser, 1975). The results demonstrated that the cross-validated communality and redundancy values are greater than zero, thus verifying adequate predictive validity in the model (Fornell and Cha, 1994; Chin, 1998; Henseler et al., 2009). All the constructs and their measures used in the study revealed adequate reliability and validity. Overall, the constructs and their respective indicators exhibited strong reliability and validity.

**TABLE 1:** Results of construct reliability and validity assessments.

Construct and indicators	Mean	STD	Item loading
*Information transparency* (AVE = 0.677, alpha = 0.842, CR = 0.894)			
We believe blockchain technology can help us disclose more transparent information, thus facilitating communication with our partners.	5.607	1.013	0.806
We believe blockchain technology can help us easily confirm historical transaction records, thus making all transactions more transparent.	5.672	1.058	0.814
We believe blockchain technology enabled-supply chain information would be transparent.	5.574	1.036	0.847
We believe the data is more transparent under the guarantee of the blockchain technology.	5.571	1.150	0.825
*Information security* (AVE = 0.742, alpha = 0.884, CR = 0.920)			
We believe the blockchain technology can enable secure data communication with our partners.	5.396	1.081	0.868
We believe blockchain technology enabled-supply chain information would be safer and more reliable.	5.382	1.135	0.860
We believe blockchain technology can keep our information more secure.	5.299	1.129	0.862
We believe blockchain technology has the potential to keep the data more secure and reliable.	5.308	1.141	0.855
*Digital leadership* (AVE = 0.722, alpha = 0.903, CR = 0.928)			
A digital leader raises awareness of the employees of the organization about the risks of the information technologies.	5.793	1.189	0.861
A digital leader raises awareness of the employees about the technologies that can be used to improve the organizational processes.	5.636	1.128	0.853
A digital leader determines required ethical behaviors for information implementations with all the stakeholders.	5.568	1.251	0.831
A digital leader plays an informative role to reduce the resistance toward innovations brought by information technologies.	5.683	1.203	0.854
A digital leader shares own experiences about technological opportunities that will increase the contributions to the colleagues for the structure of the learning organization.	5.754	1.205	0.847
*Supply chain trust* (AVE = 0.682, alpha = 0.942, CR = 0.951)			
We believe our supply chain partners usually take our interests into account when making a major decision.	5.751	1.263	0.802
We believe our supply chain partners do not seek to increase their interests at the expense of both parties.	5.683	1.188	0.822
Our supply chain partners usually keep the promises that they made to our firm.	5.852	1.144	0.839
We believe our supply chain partners can keep the promises of the cooperation made to our firm.	5.867	1.124	0.844
We believe our supply chain partners will be ready and can provide us assistance and high-quality support	5.861	1.185	0.828
Our supply chain partners usually provide us with products that have better functionality.	5.781	1.125	0.815
We believe our supply chain partners usually make decisions that are in line with the rules.	5.902	1.128	0.826
Our firm has a high level of trusting partnership with our supply chain partners.	5.766	1.144	0.829
We believe our supply chain partners will be willing and can comply with the contract.	5.902	1.117	0.829
*Supply chain flexibility* (AVE = 0.638, alpha = 0.906, CR = 0.925)			
Our supply chain is able to deal with different non-standard orders.	5.607	1.353	0.800
Our supply chain is able to offer special customer specifications.	5.639	1.211	0.788
Our supply chain is able to produce different features of products such as options, sizes, and colors.	5.746	1.219	0.814
Our supply chain is able to adjust capacity (accelerate/decelerate) in production regarding rapid customer demand changes.	5.657	1.136	0.796
Our supply chain is able to introduce large numbers of product improvements.	5.595	1.186	0.787
Our supply chain is able to offer/introduce new products for customers.	5.636	1.238	0.796
Our supply chain is able to respond to the needs and wants of the firm’s target market.	5.722	1.228	0.811

*N*, 338. AVE, average variance extracted; CR, composite reliability; STD, standard deviation.

**TABLE 2:** Correlations and discriminant validity among the constructs.

Variables	1	2	3	4	5
1. Information transparency	***0.823***				
2. Information security	0.348	***0.861***			
3. Digital leadership	0.332	0.201	***0.849***		
4. Supply chain trust	0.536	0.426	0.489	***0.826***	
5. Supply chain flexibility	0.533	0.437	0.509	0.701	***0.799***

*N*, 338. Values reported in the italicized bold diagonal text are the square root of the AVE for each construct.

### Hypotheses testing

**FIGURE 2:**
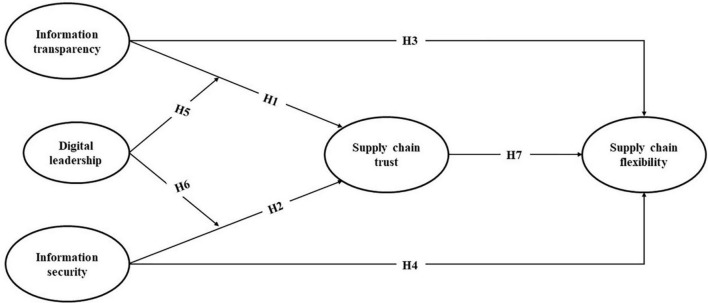
Estimated results from structural equation modeling. *N*, 338. ***p < 0.001, **p < 0.01, *p < 0.05.

Following the assessment of the measurement model, we empirically tested our hypotheses using structural equation modeling analyses. Figure 2 summarizes summarized the results of SEM analyses.

Following the approach recommended by Chin (1998), we estimated the coefficient of determination *R*^2^ and the path coefficient with their respective t-values. As reported in Figure 2, the *R*^2^ values for the two endogenous variables (i.e., supply chain trust and supply chain flexibility) exhibited adequate explanatory power for our model (0.494–0.542).

Hypotheses 1 and 2 predict a positive relationship between blockchain technology transparency, security, and supply chain trust. We tested the hypotheses using PLS to calculate the coefficients for the respective effect of blockchain technology information transparency and security. The results show a positive and statistically significant relationship between blockchain technology information transparency (*b* = 0.441, *p* < 0.01), security (*b* = 0.273, *p* < 0.01), and supply chain trust. These results support Hypotheses 1 and 2. Furthermore, we examined Hypotheses 3 and 4 by estimating the direct effect of blockchain technology information transparency and security on supply chain flexibility, respectively. The results show that blockchain technology information transparency (*b* = 0.197, *p* < 0.01) and security (*b* = 0.141, *p* < 0.05) are positively and significantly associated with supply chain flexibility, providing strong support for Hypotheses 3 and 4. Moreover, we empirically examined Hypotheses 5 and 6 by estimating the moderating effect of digital leadership on the respective contribution of blockchain technology information transparency and security to supply chain trust. As indicated in Figure 2, digital leadership negatively moderated the effect of blockchain technology information transparency on supply chain trust (*b* = -0.163, *p* < 0.01). Therefore, Hypothesis 5 is not supported. In contrast, digital leadership positively moderates the relationship between blockchain technology information security and supply chain trust (*b* = 0.175, *p* < 0.01). The results support Hypothesis 6. Finally, we test the effect of supply chain trust on supply chain flexibility. As shown in Figure 2, the path coefficient from supply chain trust to supply chain flexibility is positive and highly significant (*b* = 0.536, *p* < 0.001). Therefore, Hypothesis 7 is also supported.

While exploring the indirect effects of blockchain technology information transparency and security on supply chain flexibility *via* supply chain trust goes beyond the scope of this study, we further examined such possible indirect effects to draw more useful and important implications for scholars and managers. In this regard, we estimated the coefficients for the respective indirect effect of blockchain technology information transparency (*b* = 0.237, *p* < 0.001) and security (*b* = 0.146, *p* < 0.001) on supply chain flexibility *via* supply chain trust. The results demonstrate that blockchain technology information transparency and security indirectly affect supply chain flexibility *via* supply chain trust. In other words, these results imply that supply chain trust plays an important role in partially mediating the effect of blockchain technology information transparency and security on supply chain flexibility. We discussed the results and presented their implications in the following section.

## Discussion and conclusion

Under the circumstance that the traditional supply chain model cannot fully cope with the changes of supply chain members and supply chain risks, the necessity and urgency of firms to build a flexible supply chain are further highlighted. In the period of supply chain restructuring, how to build a flexible supply chain is an urgent problem for firms in the changing environment. Firms that want to develop a flexible supply chain in the current market must increase their flexibility. The purpose is to resist risks, restore flexibility, seize the opportunities brought by the fourth industrial revolution, and help firms quickly respond to possible risks or opportunities. Firms have been actively seeking ways to improve supply chain flexibility and integrating Industry 4.0 technology into supply chain operations has gradually become the consensus of global firm leaders. This study refers to network theory and RBV of subdividing blockchain technology into transparent information. The two characteristics of security and safety are studied, and the different effects of the two characteristics on supply chain trust and flexible supply chain are studied. Considering that the digital economy requires firms to have a certain degree of digital leadership (Petry, 2018), this study introduces digital leadership into the construction of a flexible supply chain model, explores the role of digital leadership in the construction of flexible supply chains by firms, and develops and creates a flexible supply chain model. This study lays a theoretical foundation for firms to build a flexible supply chain in the post-epidemic era. It helps firms determine how the technical characteristics of blockchain affect supply chain trust and flexible supply chain and understand the relationship between the characteristics of blockchain technology, digital leadership, supply chain trust, and flexible supply chain. It provides a valuable reference for firms to exert their digital leadership to accelerate the construction of flexible supply chain strategies in the post-epidemic era. This study can provide valuable suggestions and solutions for firms to achieve the construction of flexible supply chains. Through empirical analysis of 338 firms, this study obtains four conclusions and insights.

First, the information transparency and information security of blockchain technology contribute positively to the building of supply chain trust. Previous literature emphasized that the adoption of blockchain technology can help a firm increase customer trust (Dutta et al., 2020), fast trust (Dubey et al., 2020), and supplier trust (Meidute-Kavaliauskiene et al., 2021). This study extends this insight and proves that firms can enhance supply chain trust with the help of the information transparency and security of blockchain technology. Supply chains are inherently decentralized and complex, and supply chain node firms can easily fall into a crisis of trust. This study suggests that firms should fully use blockchain technology’s transparency and security, improve the trust of the supply chain as a whole, and create a safe and transparent supply chain environment.

Second, the information transparency and information security of blockchain also have a positive impact on supply chain flexibility. Consistent with previous literature results, adopting blockchain technology can improve supply chain resilience (Dubey et al., 2020) and guarantee firms to build a flexible supply chain (Sheel and Nath, 2019). This study confirms the positive impact of blockchain technology on the construction of a flexible supply chain, which shows that, in today’s rapidly changing information technology, the use of blockchain technology is crucial to the operation of the entire supply chain system. Firms should attach great importance to the transparency and security of blockchain technology and make full use of it to promote the transformation from a traditional supply chain to a flexible supply chain.

Third, contrary to expectations, digital leadership plays a negative moderating role in the relationship between information transparency of blockchain technology and supply chain trust. Therefore, the higher the digital leadership is, the weaker the positive relationship between information transparency of blockchain technology and supply chain trust will be. This insight is contrary to previous literature results, which believe that the higher the digital leadership is, the higher the acceptance of solving business problems through digital technologies at the forefront of Industry 4.0 (Eberl and Drews, 2021). The key reason for this expectation is that digital leadership attaches great importance to protecting firm data privacy (Outvorst et al., 2018). Since we found that digital leadership positively moderates blockchain information security and supply chain trust, the results show that digital leadership can help firms fully utilize the benefits of information security of blockchain technology to improve supply chain trust. However, our results further indicate that digital leadership may serve as a double-edged sword in moderating the relationship between blockchain technology and supply chain trust. Although high digital leadership values data security and privacy protection, it negatively impacts the relationship between information transparency and supply chain trust. Therefore, firms and their managers should be cautious about improving information transparency to build trusting relationships with their supply chain partners.

Fourth, this study found that supply chain trust positively contributes to the building of a flexible supply chain. This finding is largely consistent with previous literature which suggests that building a flexible supply chain cannot be accomplished by a firm alone but needs to achieve supply chain trust among firms (Ghode et al., 2020). This study also found that supply chain trust positively impacts the construction of a flexible supply chain and plays a mediating role between the two characteristics of blockchain technology and the building of a flexible supply chain, thereby providing insights into the relationships between information transparency and security of blockchain technology, supply chain trust, and supply chain flexibility. This implies that a firm needs to adopt blockchain technology for them to build trusting relationships with its partners and develop a flexible supply chain, especially in uncertain situations. It is thus necessary for executives to pay close attention to the important role of building trust-based relationships in helping the firm develop a flexible supply chain.

Like all studies, this study has some limitations which can offer important opportunities for future research. First, this study only empirically examined our claims using Chinese firms that widely use blockchain technology, which may limit the generalizability of our findings. Future studies should expand the research scope to additional research contexts to increase the generalizability of our results. Second, while we believe blockchain technology is widely adopted in many different industries, we primarily focused on firms operating in the manufacturing sector. Future studies are encouraged to scrutinize our core expectations and findings in other alternative sample settings, possibly incorporating a greater number of industries. Third, as blockchain technology is developing iteratively, it may have additional important characteristics such as information immutability and traceability except for information transparency and security. Further research should incorporate additional important characteristics of blockchain technology into the conceptual model and explore their potential effects on the building of supply chain trust and flexibility. In addition, making information transparent may not guarantee better firm performance. In particular, as pointed out in the literature, blockchain technology is not yet mature, whether it can bring value to business processes remains unclear, and many firms are thus taking a wait-and-see attitude toward blockchain technology (Swan, 2015; Liang et al., 2021). Future researchers thus need to explore whether trusting relationships and flexibility achieved by using blockchain technology in the supply chain can contribute to improved firm performance. Finally, going beyond context-specific, industry-specific, and blockchain technology characteristic-specific, future researchers should also engage with additional firm-specific heterogeneity, such as dynamic capabilities and competencies in the links between different characteristics of blockchain technology and supply chain trust.

## Data availability statement

The raw data supporting the conclusions of this article will be made available by the authors, without undue reservation.

## Author contributions

Both authors listed have made a substantial, direct, and intellectual contribution to the work, and approved it for publication.
